# Reduction of pin tract infections during external fixation using cadexomer iodine

**DOI:** 10.1186/s40634-020-00305-y

**Published:** 2020-11-07

**Authors:** Mylène P. Jansen, Nienke van Egmond, Esmee C. Kester, Simon C. Mastbergen, Floris P. J. G. Lafeber, Roel J. H. Custers

**Affiliations:** 1 Department of Rheumatology & Clinical Immunology, University Medical Center Utrecht, Heidelberglaan 100 (G02.228), 3584CX Utrecht, The Netherlands; 2 Department of Orthopedic Surgery, University Medical Center Utrecht, Utrecht, The Netherlands

**Keywords:** External fixation, Infection, Pin tract, Joint distraction, Cadexomer iodine, Regular care, Prevention, Ointment, Pin track

## Abstract

**Purpose:**

Knee joint distraction (KJD) is a joint-preserving treatment for younger osteoarthritis patients. KJD has shown positive results in regular care, but the external fixation frame often caused pin tract skin infections. Therefore, the use of cadexomer iodine was included in the wound care protocol. The goal of this cross-sectional study was to evaluate whether use of this ointment reduced the number of patients with infections during KJD treatment.

**Methods:**

Patients treated with KJD in regular care were included if they gave consent for use of their data and completed treatment with the newest distraction device before 2020. All patients followed a wound care protocol, which since March 2019 included using cadexomer iodine ointment. The number of patients experiencing pin tract infections was compared between patients who did (March 2019–December 2019) and did not (November 2017–March 2019) use the ointment.

**Results:**

Sixty-seven patients were included; 34 patients used cadexomer iodine and 33 patients did not. Patient who did not use cadexomer iodine experienced twice as many infections (64% vs 32%;*p* = 0.010). There was a significant difference in the number of patients with serious infections, requiring more antibiotics than the standard 7-day oral antibiotics (30% without vs 6% with cadexomer iodine; *p* = 0.009).

**Conclusions:**

The use of cadexomer iodine ointment during KJD results in a significant reduction of the number of patients experiencing pin tract infections during treatment. Use of this ointment should be considered standard protocol during KJD treatment and could be of value in general external fixator usage as well.

## Background

Knee joint distraction (KJD) is a joint-preserving treatment for younger (< 65 years) patients with severe knee osteoarthritis (OA). KJD aims to postpone total knee arthroplasty (TKA) and decrease the chance of a revision TKA later in life [7].

In KJD, the tibia and femur are placed at 5 mm distance for 6 weeks using an external fixation frame, fixed to the bones using 8 trans-cutaneous half pins. KJD has shown clinical benefit similar to TKA or osteotomy, as well as cartilage repair activity [7, 9, 10, 22–25]. Effects can last for years, evaluated up to 9 years thus far [13]. Despite positive results that were observed in trials and regular care, the treatment can be a 6-week burden for patients when pin tract skin infections occur [11]. Pin tract infections are often seen in external fixation devices, and while a small number of studies have been published on how to prevent these infections, literature on this topic is limited [1, 8, 14, 16, 17]. Although in KJD the infections did not seem to have an influence on the patients’ clinical benefit, prevention could decrease the burden of this promising treatment [11]. Updating the wound care protocol (see: Methods) in between clinical trials revealed a positive effect in decreasing infections, reducing pin tract infections from 85% to 57% of patients [11]. However, further reduction was clearly desirable [9]. Therefore, the use of cadexomer iodine ointment was included in the KJD wound care protocol in regular care. The objective of this study was to evaluate whether using cadexomer iodine ointment reduced the number of patients with pin tract infections during KJD treatment.

## Methods

### Patients

In the UMC Utrecht, knee OA patients with an indication for TKA, but younger than 65 years old, were offered KJD treatment in regular care. Specific considerations and criteria for KJD treatment in regular care have been described previously [11].

As standard procedure, all patients treated at the department of orthopedics are asked written consent for use of their anonymized data for future research purposes (protocol number 17–005). Ethical approval for this study was waived by the medical ethical review board of the University Medical Center Utrecht (protocol number 20–128/C). While KJD has been performed in regular care since 2014, a new dedicated distraction device (KneeReviver®; ArthroSave, Culemborg, The Netherlands) was introduced in November 2017, which was developed to better facilitate pin care and showed a significant reduction in pin tract infections [12]. To prevent bias, only patients who received the full KJD treatment with the KneeReviver® and had their frame removed before 2020 were included in the current cross-sectional study. All included patients gave written informed consent.

### Treatment

**Fig. 1:**
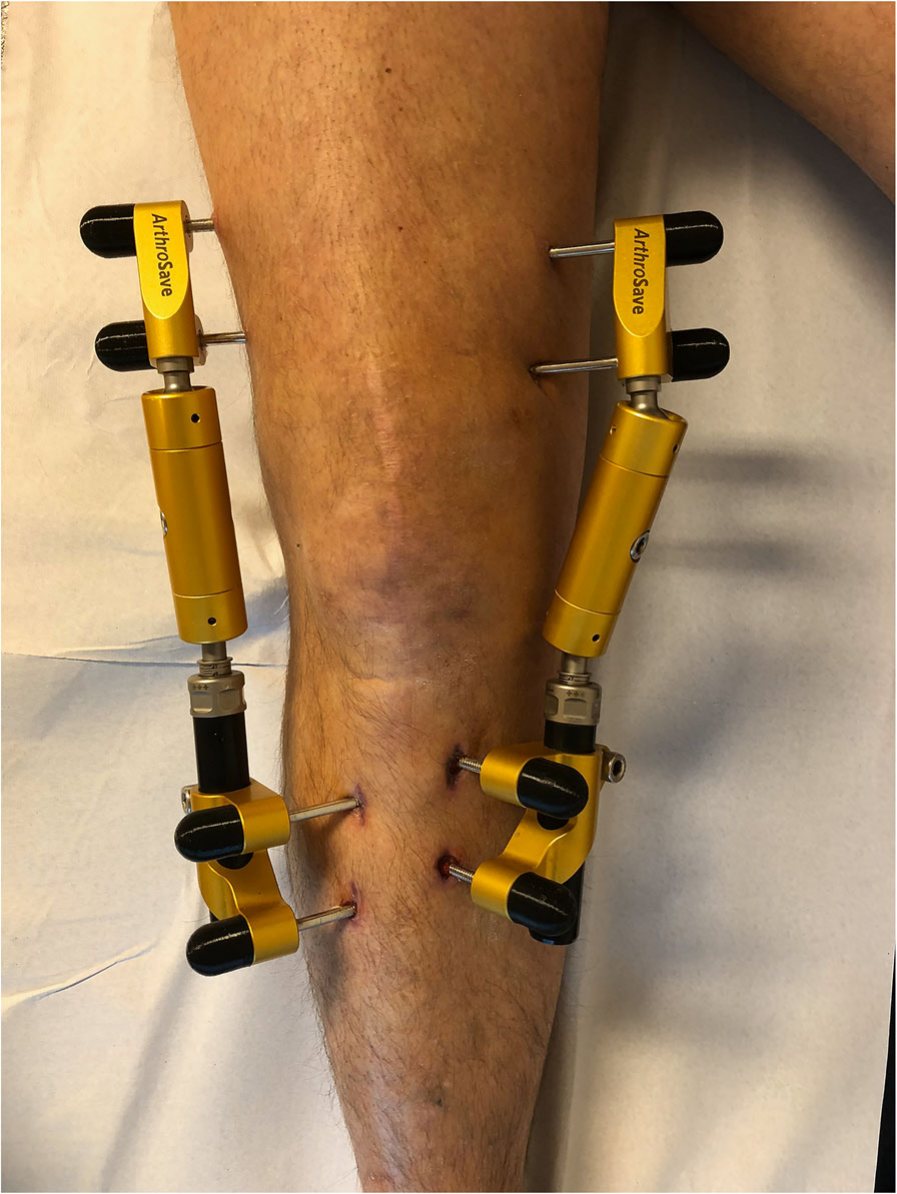
The external fixation frame used for knee joint distraction treatment

The treatment protocol in regular care has been extensively described [11]. In short, the tibia and femur were distracted for at least 5 mm for 6 to 7 weeks, using an external fixation frame (KneeReviver®) that consisted of 2 distraction tubes, 1 placed medially and 1 laterally of the knee joint. The tubes were fixed to the bones using 8 trans-cutaneous half pins, placed in pairs at 4 locations (medial/lateral and tibia/femur), as shown in Fig. 1. Distraction was obtained gradually over the course of 3 days, and after radiographic confirmation, patients were discharged from the hospital with a standard prescription for 7 days of oral antibiotics (flucloxacillin; 3 times per day 500 mg) only to be used in case of infection (not as prophylaxis). In case a patient suspected a pin tract infection, they consulted their physician and based on the physician’s judgement started their 7-day antibiotic course. If this standard course was not enough or more infections occurred during the distraction period or shortly thereafter, patients received additional antibiotic courses as necessary. During treatment, full weight-bearing was encouraged, supported by crutches if necessary.

After 4 weeks patients returned to the outpatient clinic for a general evaluation, and after 6 to 7 weeks the distraction frame was removed in daycare.

### Cadexomer iodine (Iodosorb®, Smith & Nephew)

Since March 2019, patients treated with KJD receive antimicrobial ointment to use on pin tracts during the distraction period. Iodosorb® (Smith & Nephew, Watford, United Kingdom) ointment consists of small cadexomer (polysaccharide) beads containing 0.9% iodine that can absorb wound exudate, pus and debris [3, 18]. The absorption causes the beads to swell, allowing a sustained release of iodine. As more iodine is released, the color gradually changes from brown to white/gray, indicating the ointment is no longer effective and wound care should be performed.

### Wound care protocol

Except for the use of Iodosorb®, the advised wound care protocol was identical for all patients. Patients were instructed to perform the following wound care every 1 to 3 days: first, the distraction frame is cleaned using non-sterile water (for example in the shower) and the gauze around all pins is removed. If the patient used Iodosorb®, the old ointment is removed from the wounds. The skin around the pins is massaged, freeing it from the pin and causing any accumulation of exudate to surface. After, the pins are cleaned using 70% alcohol, moving from the skin upwards. The skin around the pins is cleaned by dabbing it with chlorhexidine 0.5% (in alcohol 70%), using clean gauze. If the patient is using Iodosorb®, fresh ointment is subsequently reapplied to the wounds; if the wounds are clean and dry, application is not needed. Finally, clean gauze is applied around the pins and fixed with plasters.

After removal of the KneeReviver, Iodosorb® was not applied anymore.

### Statistical analyses

Patients who used Iodosorb® during their KJD treatment (March 2019 – December 2019) were compared with patients who did not use Iodosorb® during treatment (November 2017 – March 2019). Baseline age, sex, BMI, diabetes mellitus, smoking status and treated leg (left/right) were compared between the 2 groups using independent two-tailed t-tests for continuous variables and chi-square tests for nominal variables. Diabetes mellitus and smoking status were included because they, like age and sex, are known risk factors for infections during fixation [4, 15]. All data was extracted from patients’ electronic records; no missing data was expected since all data was required before treatment could be performed.

Outcome parameters were the number of patients requiring antibiotics for pin tract infections, the number of patients requiring more than 1 standard 7-day oral antibiotic course (indicating a more serious infection), and the number of patients with infections after frame removal. All 3 outcome parameters were compared between groups using chi-square tests. *P*-values < 0.05 were considered statistically significant. IBM SPSS Statistics version 25 (IBM Corp; Armonk, NY) was used for all statistical analyses.

## Results

### Patients

Before 2020, a total of 73 patients were treated with the latest distraction device, of whom 68 gave permission for use of their data. In 1 patient full treatment was not carried out (frame was removed within a week because of pain), while the other 67 patients received full KJD treatment. Of these, 34 patients used Iodosorb® during treatment, while the other 33 patients did not. The baseline characteristics of both groups are shown in Table 1, showing no significant differences between groups. There was no missing data.

**Table 1:** Baseline characteristics of knee joint distraction patients with or without cadoxemer iodine (Iodosorb®)

	Without Iodosorb® (***n*** = 33)	With Iodosorb® (***n*** = 34)	***p***-value
**Age, mean ± SD**	52.0 ± 7.0	52.9 ± 7.6	0.624
**Male gender, n (%)**	14 (42)	19 (56)	0.271
**BMI, mean ± SD**	27.0 ± 3.0	27.9 ± 2.8	0.259
**Diabetes mellitus, n (%)**	1 (3)	1 (3)	0.983
**Smoking status, n (%)**			0.921
- **Never**	20 (61)	19 (56)	
- **Former**	12 (36)	14 (41)	
- **Current**	1 (3)	1 (3)	
**Left leg, n (%)**	11 (33)	16 (47)	0.252

### Infections

The number of patients who experienced infections during and after treatment are shown in Table 2 for both groups. During treatment, patients who did not use Iodosorb® experienced twice as many infections as patients who used the ointment (64% vs 32%; *p* = 0.010).

**Table 2:** Infections during and after treatment with and without use of cadexomer iodine (Iodosorb®) during treatment

	Without Iodosorb® (***n*** = 33)	With Iodosorb® (***n*** = 34)	***p***-value
**Patients with pin tract infections during treatment, n (%)**	21 (64)	11 (32)	**0.010**
**Patients with > 1 seven-day antibiotics course, n (%)**	10 (30)	2 (6)	**0.009**
**Patients with infections after treatment, n (%)**	2 (6)	3 (9)	0.667

Also, there was a significant difference in the number of patients with more serious infections, requiring more antibiotics than the standard 7-day antibiotic prescription (30% without Iodosorb® vs 6% with Iodosorb®; *p* = 0.009). In all cases, the additional antibiotics consisted of multiple courses or 1 longer course of oral antibiotics; none of the patients required hospital admission or intravenous antibiotics during treatment.

The number of patients experiencing infections after frame removal did not differ significantly between groups (6% without Iodosorb® vs 9% with Iodosorb®; *p* = 0.667). After frame removal, in the group without Iodosorb®, 1 patient received intravenous antibiotics while admitted to the hospital because of suspected osteomyelitis, and the other 1 received 1 7-day course of oral antibiotics for pin tract infection. In the group with Iodosorb®, after frame removal, 1 patient received intravenous antibiotics while admitted to the hospital because of suspected osteomyelitis, a second patient was admitted to the hospital and treated with intravenous antibiotics for a postoperative abscess and a third patient received 1 standard course of oral antibiotics because a pin tract wound was not completely healed.

## Discussion

The most important finding of the present study was that for patients treated with KJD, incorporating the use of cadexomer iodine ointment in the wound care protocol significantly reduces the prevalence of pin tract infections. The number of patients experiencing pin tract infections decreased with 50% by using Iodosorb®. This is a clinically relevant reduction that implicates a significant decrease in treatment burden of patients. An even bigger difference was seen in the number of patients requiring more than a 7-day course of oral antibiotics. The number of patients with these more frequent or serious infections was reduced by 80%. It can be expected that this influences the patient’s general physical and mental health during treatment. The use of cadexomer iodine during KJD treatment did not seem to have an effect on the number of patients experiencing infections after removal of the distraction frame. This may be related to ceasing application of the ointment too early.

None of the patients in either group required hospital admission and intravenous antibiotics during treatment. This is a remarkable difference with the previously reported complications experienced in KJD patients treated in regular care, where intravenous antibiotics were necessary for 14% of patients [11]. The fact that in the current study this number was reduced to zero does not seem to be a result of cadexomer iodine use, but may be because of the use of the ArthroSave KneeReviver® frame as compared to the Stryker Dynamic Monotubes used in previous studies, considered by patients to be advantageous with respect to wound care [12].

The number of patients experiencing pin tract infections in this study was based on how many patients required antibiotics. In regular care, when patients have complaints of their pin tract wound and suspect an infection, they consult their physician. If the physician decides that it is an infection, based on the patient’s complaints of pain around the pin tract as well as redness, warmth and pus presence, the patient can start their prescription of antibiotics. As a result, these infections are not confirmed by, for example, positive bacterial cultures. Although it has been shown that swab cultures in pin tract infections are not very helpful [6], it is possible that some patients started antibiotics without actually having a pin tract infection, in which case the amount of pin tract infections might be lower than presented in this study. While this was a limitation of the current study, all patients taking antibiotics experienced infection-like symptoms and received antibiotics according to regular care protocol, so the significant reduction experienced after use of cadexomer iodine is clearly relevant in clinical practice and has direct implications for both patient wellbeing and general antibiotic use. It may, however, have been useful to not only compare the number of patients experiencing infections, but also the number of infected pins, as is often done in other studies. We did not collect this data, or different outcomes such as systemic biomarker levels to evaluate the effect of the ointment on general physiological functions, as this was a retrospective analysis.

Another limitation of the current study was that it was not set up as a randomized controlled trial. Ideally, patients receiving cadexomer iodine would be compared to patients using a placebo ointment in a randomized controlled trial. Nevertheless, the 2 patient groups seem similar and do not show any statistically significant differences in baseline characteristics, including known risk factors for infections during fixation. At present, a randomized trial while knowing the difference in infections between both groups would be ethically unsound. However, an interesting future study may be a randomized controlled trial comparing Iodosorb® to one or more other agents or methods for pin tract infection prevention.

Despite significant reductions in patients with infections, still a third of KJD patients experience pin tract infections. Further reduction of pin tract infections, which might be achieved by additional changes in the surgical technique, equipment (pins) or wound care protocol, is required to further reduce antibiotic use and the patients’ treatment burden during KJD. Literature on preventing pin tract infections associated with external fixators is limited, and studies that evaluated factors such as cleansing solutions, prophylactic antibiotic use, different types of dressings, pin coating, and pin care frequency generally found no significant effects [5, 8, 14, 16, 20]. However, combined with cadexomer iodine use, implementing other changes might result in a further reduction of pin tract infections. Although it was previously shown infections do not have an influence on clinical benefit, and patients undergoing TKA several years after KJD did not experience additional complications or decreased clinical benefit, prevention of pin tract infections could still have positive effects in decreasing the patients’ treatment burden during the fixation period [11, 21].

While the use of cadexomer iodine in patients has been evaluated and shown positive results, these studies were all performed in patients with ulcers [2, 18, 19]. Based on the significant results found in the current study, the use of cadexomer iodine in other treatments that use external fixation frames could be considered and evaluated as well, as it is likely that these results are not specific to only KJD.

In conclusion, the use of cadexomer iodine ointment during KJD results in a significant reduction of the number of patients experiencing pin tract infections during treatment in regular care. Use of this ointment may be considered as standard protocol during KJD treatment and could be of value in general external fixator usage as well.

## Data Availability

The datasets used during the current study are not publicly available due to ethical restrictions related to participant consent, but are available from the corresponding author on reasonable request.

## Abbreviations

KJD: Knee joint distraction
OA: Osteoarthritis
TKA: Total knee arthroplasty
